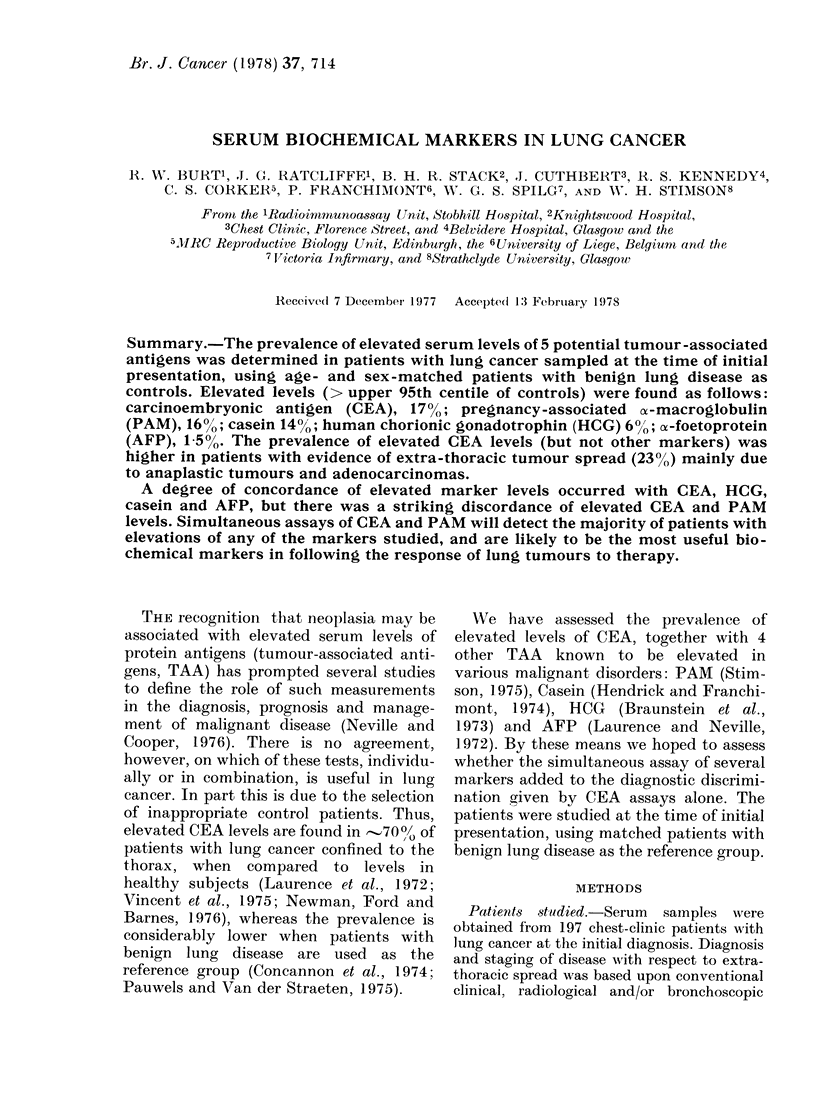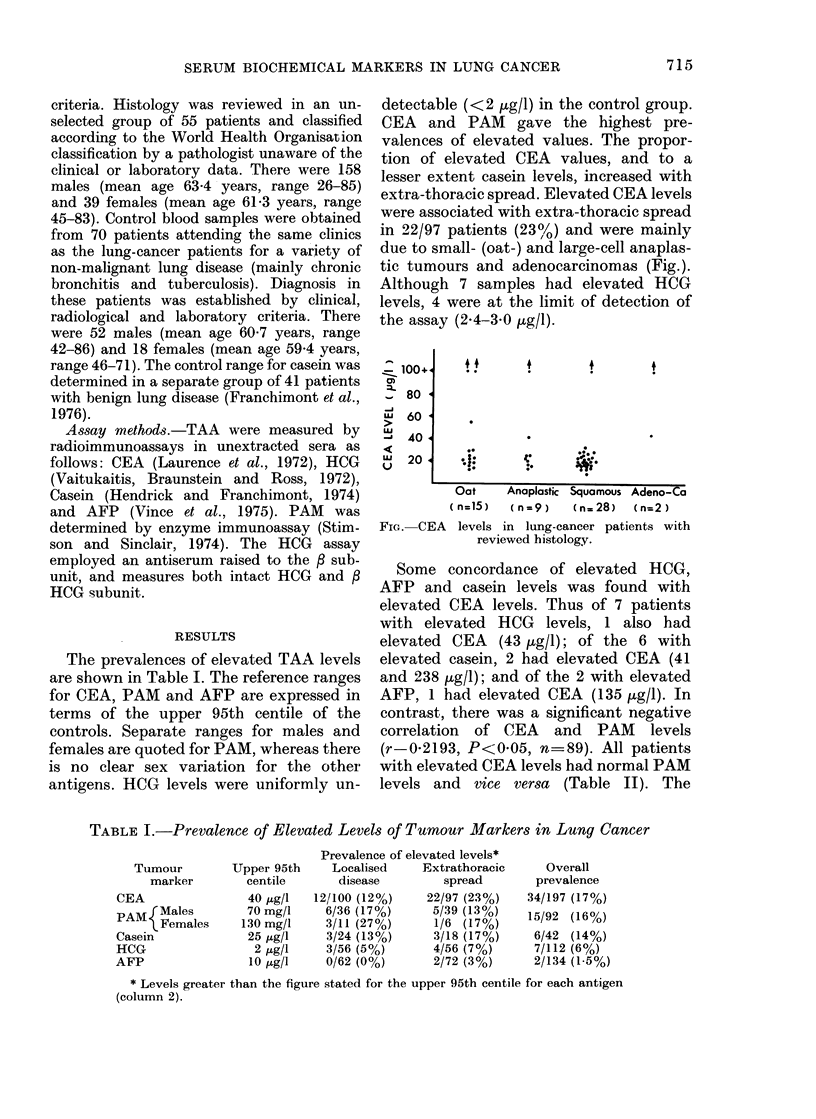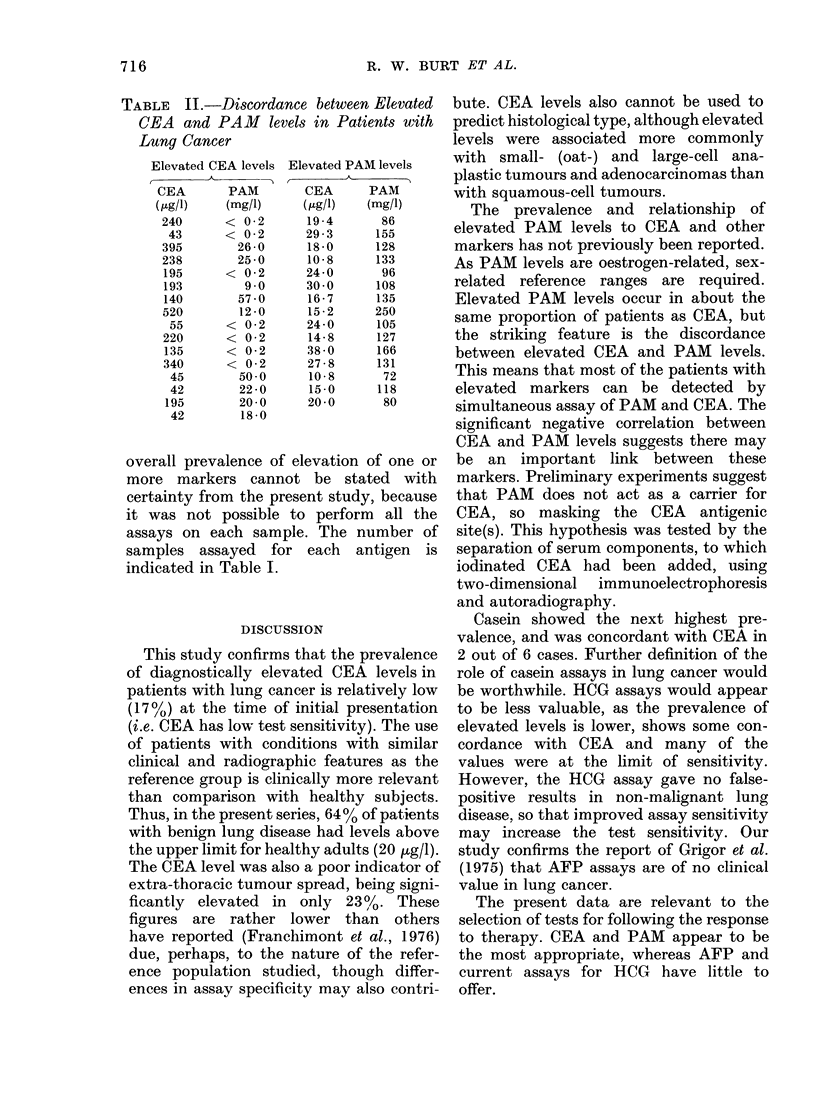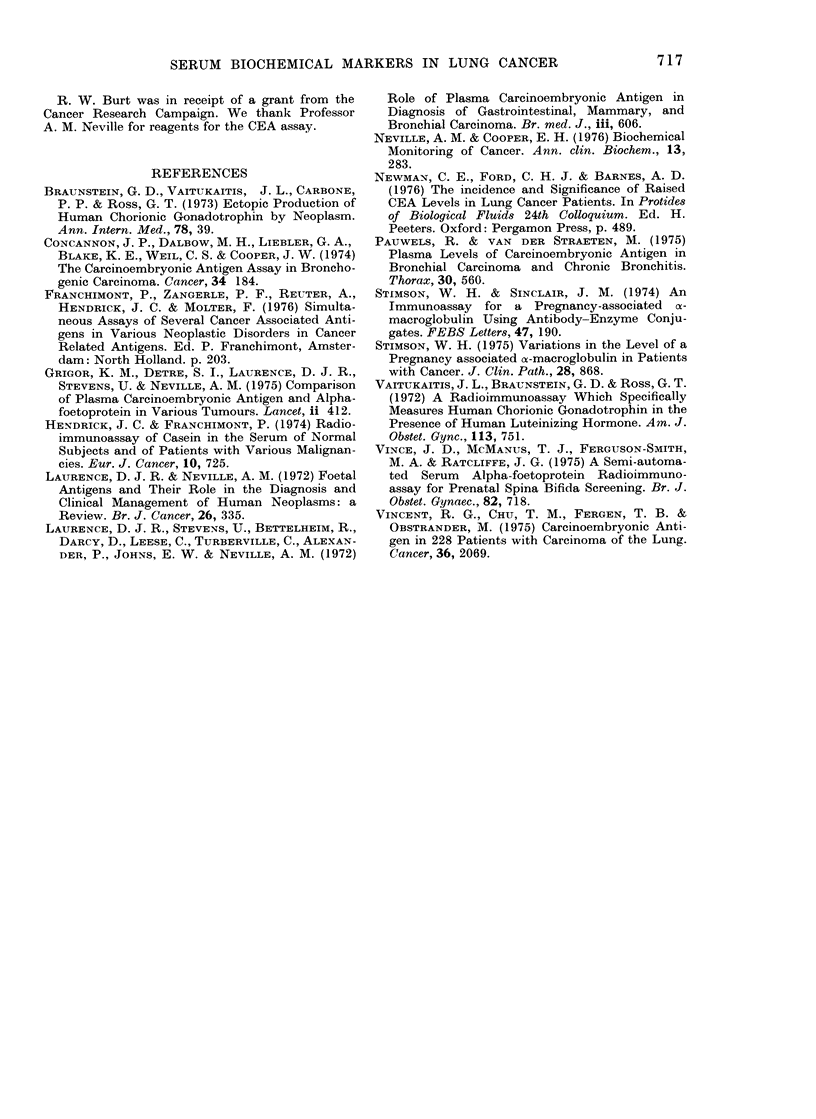# Serum biochemical markers in lung cancer.

**DOI:** 10.1038/bjc.1978.108

**Published:** 1978-05

**Authors:** R. W. Burt, J. G. Ratcliffe, B. H. Stack, J. Cuthbert, R. S. Kennedy, C. S. Corker, P. Franchimont, W. G. Spilg, W. H. Stimson

## Abstract

The prevalence of elevated serum levels of 5 potential tumour-associated antigens was determined in patients with lung cancer sampled at the time of initial presentation, using age- and sex-matched patients with benign lung disease as controls. Elevated levels (greater than upper 95th centile of controls) were found as follows: carcinoembryonic antigen (CEA), 17%; pregnancy-associated alpha-macroglobulin (PAM), 16%; casein 14%; human chorionic gonadotrophin (HCG) 6%; alpha-foetoprotein (AFP), 1.5%. The prevalence of elevated CEA levels (but not other markers) was higher in patients with evidence of extra-thoracic tumour spread (23%) mainly due to anaplastic tumours and adenocarcinomas. A degree of concordance of elevated marker levels occurred with CEA, HCG, casein and AFP, but there was a striking discordance of elevated CEA and PAM levels. Simultaneous assays of CEA and PAM will detect the majority of patients with elevations of any of the markers studied, and are likely to be the most useful biochemical markers in following the response of lung tumours to therapy.


*Br. J. Cancer* (1978) **37**, 714

### SERUM BIOCHEMICAL MARKERS IN LUNG CANCER

R. W. BURT[1], J. G. RATCLIFFE[1], B. H. R. STACK[2], J. CUTHBERT[3], R. S. KENNEDY[4], C. S. CORKER[5], P. FRANCHIMONT[6], W. G. S. SPILG[7], AND W. H. STIMSON[8]

*From the [1]Radioimmunoassay Unit, Stobhill Hospital, [2]Knightswood Hospital, [3]Chest Clinic, Florence Street, and [4]Belvidere Hospital, Glasgow and the [5]MRC Reproductive Biology Unit, Edinburgh, the [6]University of Liege, Belgium and the [7]Victoria Infirmary, and [8]Strathclyde University, Glasgow*

Received 7 December 1977 Accepted 13 February 1978

**Summary.—The prevalence of elevated serum levels of 5 potential tumour-associated antigens was determined in patients with lung cancer sampled at the time of initial presentation, using age- and sex-matched patients with benign lung disease as controls. Elevated levels (> upper 95th centile of controls) were found as follows: carcinoembryonic antigen (CEA), 17%; pregnancy-associated α-macroglobulin (PAM), 16%; casein 14%; human chorionic gonadotrophin (HCG) 6%; α-foetoprotein (AFP), 1·5%. The prevalence of elevated CEA levels (but not other markers) was higher in patients with evidence of extra-thoracic tumour spread (23%) mainly due to anaplastic tumours and adenocarcinomas.**

**A degree of concordance of elevated marker levels occurred with CEA, HCG, casein and AFP, but there was a striking discordance of elevated CEA and PAM levels. Simultaneous assays of CEA and PAM will detect the majority of patients with elevations of any of the markers studied, and are likely to be the most useful biochemical markers in following the response of lung tumours to therapy.**

THE recognition that neoplasia may be associated with elevated serum levels of protein antigens (tumour-associated antigens, TAA) has prompted several studies to define the role of such measurements in the diagnosis, prognosis and management of malignant disease (Neville and Cooper, 1976). There is no agreement, however, on which of these tests, individually or in combination, is useful in lung cancer. In part this is due to the selection of inappropriate control patients. Thus, elevated CEA levels are found in ~70% of patients with lung cancer confined to the thorax, when compared to levels in healthy subjects (Laurence *et al.*, 1972; Vincent *et al.*, 1975; Newman, Ford and Barnes, 1976), whereas the prevalence is considerably lower when patients with benign lung disease are used as the reference group (Concannon *et al.*, 1974; Pauwels and Van der Straeten, 1975).

We have assessed the prevalence of elevated levels of CEA, together with 4 other TAA known to be elevated in various malignant disorders: PAM (Stimson, 1975), Casein (Hendrick and Franchimont, 1974), HCG (Braunstein *et al.*, 1973) and AFP (Laurence and Neville, 1972). By these means we hoped to assess whether the simultaneous assay of several markers added to the diagnostic discrimination given by CEA assays alone. The patients were studied at the time of initial presentation, using matched patients with benign lung disease as the reference group.

### METHODS

*Patients studied.*—Serum samples were obtained from 197 chest-clinic patients with lung cancer at the initial diagnosis. Diagnosis and staging of disease with respect to extra-thoracic spread was based upon conventional clinical, radiological and/or bronchoscopic criteria. Histology was reviewed in an unselected group of 55 patients and classified according to the World Health Organisation classification by a pathologist unaware of the clinical or laboratory data. There were 158 males (mean age 63·4 years, range 26–85) and 39 females (mean age 61·3 years, range 45–83). Control blood samples were obtained from 70 patients attending the same clinics as the lung-cancer patients for a variety of non-malignant lung disease (mainly chronic bronchitis and tuberculosis). Diagnosis in these patients was established by clinical, radiological and laboratory criteria. There were 52 males (mean age 60·7 years, range 42–86) and 18 females (mean age 59·4 years, range 46–71). The control range for casein was determined in a separate group of 41 patients with benign lung disease (Franchimont *et al.*, 1976).

*Assay methods.*—TAA were measured by radioimmunoassays in unextracted sera as follows: CEA (Laurence *et al.*, 1972), HCG (Vaitukaitis, Braunstein and Ross, 1972), Casein (Hendrick and Franchimont, 1974) and AFP (Vince *et al.*, 1975). PAM was determined by enzyme immunoassay (Stimson and Sinclair, 1974). The HCG assay employed an antiserum raised to the $\beta$ subunit, and measures both intact HCG and $\beta$ HCG subunit.

### RESULTS

The prevalences of elevated TAA levels are shown in Table I. The reference ranges for CEA, PAM and AFP are expressed in terms of the upper 95th centile of the controls. Separate ranges for males and females are quoted for PAM, whereas there is no clear sex variation for the other antigens. HCG levels were uniformly undetectable (<2 $\mu$g/l) in the control group. CEA and PAM gave the highest prevalences of elevated values. The proportion of elevated CEA values, and to a lesser extent casein levels, increased with extra-thoracic spread. Elevated CEA levels were associated with extra-thoracic spread in 22/97 patients (23%) and were mainly due to small- (oat-) and large-cell anaplastic tumours and adenocarcinomas (Fig.). Although 7 samples had elevated HCG levels, 4 were at the limit of detection of the assay (2·4–3·0 $\mu$g/l).

FIG.—CEA levels in lung-cancer patients with reviewed histology.

Some concordance of elevated HCG, AFP and casein levels was found with elevated CEA levels. Thus of 7 patients with elevated HCG levels, 1 also had elevated CEA (43 $\mu$g/l); of the 6 with elevated casein, 2 had elevated CEA (41 and 238 $\mu$g/l); and of the 2 with elevated AFP, 1 had elevated CEA (135 $\mu$g/l). In contrast, there was a significant negative correlation of CEA and PAM levels ($r$—0·2193, $P$<0·05, $n$=89). All patients with elevated CEA levels had normal PAM levels and *vice versa* (Table II). The

TABLE I.—*Prevalence of Elevated Levels of Tumour Markers in Lung Cancer*

| Tumour marker | | Upper 95th centile | Prevalence of elevated levels* Localised disease | Extrathoracic spread | Overall prevalence |
|---|---|---|---|---|---|
| CEA | | 40 $\mu$g/l | 12/100 (12%) | 22/97 (23%) | 34/197 (17%) |
| PAM | Males | 70 mg/l | 6/36 (17%) | 5/39 (13%) | 15/92 (16%) |
| | Females | 130 mg/l | 3/11 (27%) | 1/6 (17%) | |
| Casein | | 25 $\mu$g/l | 3/24 (13%) | 3/18 (17%) | 6/42 (14%) |
| HCG | | 2 $\mu$g/l | 3/56 (5%) | 4/56 (7%) | 7/112 (6%) |
| AFP | | 10 $\mu$g/l | 0/62 (0%) | 2/72 (3%) | 2/134 (1·5%) |

* Levels greater than the figure stated for the upper 95th centile for each antigen (column 2).

TABLE II.—*Discordance between Elevated CEA and PAM levels in Patients with Lung Cancer*

| Elevated CEA levels | | Elevated PAM levels | |
|---|---|---|---|
| CEA (μg/l) | PAM (mg/l) | CEA (μg/l) | PAM (mg/l) |
| 240 | < 0·2 | 19·4 | 86 |
| 43 | < 0·2 | 29·3 | 155 |
| 395 | 26·0 | 18·0 | 128 |
| 238 | 25·0 | 10·8 | 133 |
| 195 | < 0·2 | 24·0 | 96 |
| 193 | 9·0 | 30·0 | 108 |
| 140 | 57·0 | 16·7 | 135 |
| 520 | 12·0 | 15·2 | 250 |
| 55 | < 0·2 | 24·0 | 105 |
| 220 | < 0·2 | 14·8 | 127 |
| 135 | < 0·2 | 38·0 | 166 |
| 340 | < 0·2 | 27·8 | 131 |
| 45 | 50·0 | 10·8 | 72 |
| 42 | 22·0 | 15·0 | 118 |
| 195 | 20·0 | 20·0 | 80 |
| 42 | 18·0 | | |

overall prevalence of elevation of one or more markers cannot be stated with certainty from the present study, because it was not possible to perform all the assays on each sample. The number of samples assayed for each antigen is indicated in Table I.

### DISCUSSION

This study confirms that the prevalence of diagnostically elevated CEA levels in patients with lung cancer is relatively low (17%) at the time of initial presentation (*i.e.* CEA has low test sensitivity). The use of patients with conditions with similar clinical and radiographic features as the reference group is clinically more relevant than comparison with healthy subjects. Thus, in the present series, 64% of patients with benign lung disease had levels above the upper limit for healthy adults (20 μg/l). The CEA level was also a poor indicator of extra-thoracic tumour spread, being significantly elevated in only 23%. These figures are rather lower than others have reported (Franchimont *et al.*, 1976) due, perhaps, to the nature of the reference population studied, though differences in assay specificity may also contribute. CEA levels also cannot be used to predict histological type, although elevated levels were associated more commonly with small- (oat-) and large-cell anaplastic tumours and adenocarcinomas than with squamous-cell tumours.

The prevalence and relationship of elevated PAM levels to CEA and other markers has not previously been reported. As PAM levels are oestrogen-related, sex-related reference ranges are required. Elevated PAM levels occur in about the same proportion of patients as CEA, but the striking feature is the discordance between elevated CEA and PAM levels. This means that most of the patients with elevated markers can be detected by simultaneous assay of PAM and CEA. The significant negative correlation between CEA and PAM levels suggests there may be an important link between these markers. Preliminary experiments suggest that PAM does not act as a carrier for CEA, so masking the CEA antigenic site(s). This hypothesis was tested by the separation of serum components, to which iodinated CEA had been added, using two-dimensional immunoelectrophoresis and autoradiography.

Casein showed the next highest prevalence, and was concordant with CEA in 2 out of 6 cases. Further definition of the role of casein assays in lung cancer would be worthwhile. HCG assays would appear to be less valuable, as the prevalence of elevated levels is lower, shows some concordance with CEA and many of the values were at the limit of sensitivity. However, the HCG assay gave no false-positive results in non-malignant lung disease, so that improved assay sensitivity may increase the test sensitivity. Our study confirms the report of Grigor *et al.* (1975) that AFP assays are of no clinical value in lung cancer.

The present data are relevant to the selection of tests for following the response to therapy. CEA and PAM appear to be the most appropriate, whereas AFP and current assays for HCG have little to offer.

R. W. Burt was in receipt of a grant from the Cancer Research Campaign. We thank Professor A. M. Neville for reagents for the CEA assay.

### REFERENCES

BRAUNSTEIN, G. D., VAITUKAITIS, J. L., CARBONE, P. P. & ROSS, G. T. (1973) Ectopic Production of Human Chorionic Gonadotrophin by Neoplasm. *Ann. Intern. Med.*, **78**, 39.

CONCANNON, J. P., DALBOW, M. H., LIEBLER, G. A., BLAKE, K. E., WEIL, C. S. & COOPER, J. W. (1974) The Carcinoembryonic Antigen Assay in Bronchogenic Carcinoma. *Cancer*, **34** 184.

FRANCHIMONT, P., ZANGERLE, P. F., REUTER, A., HENDRICK, J. C. & MOLTER, F. (1976) Simultaneous Assays of Several Cancer Associated Antigens in Various Neoplastic Disorders in Cancer Related Antigens. Ed. P. Franchimont, Amsterdam: North Holland. p. 203.

GRIGOR, K. M., DETRE, S. I., LAURENCE, D. J. R., STEVENS, U. & NEVILLE, A. M. (1975) Comparison of Plasma Carcinoembryonic Antigen and Alphafoetoprotein in Various Tumours. *Lancet*, **ii** 412.

HENDRICK, J. C. & FRANCHIMONT, P. (1974) Radioimmunoassay of Casein in the Serum of Normal Subjects and of Patients with Various Malignancies. *Eur. J. Cancer*, **10**, 725.

LAURENCE, D. J. R. & NEVILLE, A. M. (1972) Foetal Antigens and Their Role in the Diagnosis and Clinical Management of Human Neoplasms: a Review. *Br. J. Cancer*, **26**, 335.

LAURENCE, D. J. R., STEVENS, U., BETTELHEIM, R., DARCY, D., LEESE, C., TURBERVILLE, C., ALEXANDER, P., JOHNS, E. W. & NEVILLE, A. M. (1972) Role of Plasma Carcinoembryonic Antigen in Diagnosis of Gastrointestinal, Mammary, and Bronchial Carcinoma. *Br. med. J.*, **iii**, 606.

NEVILLE, A. M. & COOPER, E. H. (1976) Biochemical Monitoring of Cancer. *Ann. clin. Biochem.*, **13**, 283.

NEWMAN, C. E., FORD, C. H. J. & BARNES, A. D. (1976) The incidence and Significance of Raised CEA Levels in Lung Cancer Patients. In *Protides of Biological Fluids 24th Colloquium*. Ed. H. Peeters. Oxford: Pergamon Press, p. 489.

PAUWELS, R. & VAN DER STRAETEN, M. (1975) Plasma Levels of Carcinoembryonic Antigen in Bronchial Carcinoma and Chronic Bronchitis. *Thorax*, **30**, 560.

STIMSON, W. H. & SINCLAIR, J. M. (1974) An Immunoassay for a Pregnancy-associated α-macroglobulin Using Antibody–Enzyme Conjugates. *FEBS Letters*, **47**, 190.

STIMSON, W. H. (1975) Variations in the Level of a Pregnancy associated α-macroglobulin in Patients with Cancer. *J. Clin. Path.*, **28**, 868.

VAITUKAITIS, J. L., BRAUNSTEIN, G. D. & ROSS, G. T. (1972) A Radioimmunoassay Which Specifically Measures Human Chorionic Gonadotrophin in the Presence of Human Luteinizing Hormone. *Am. J. Obstet. Gync.*, **113**, 751.

VINCE, J. D., MCMANUS, T. J., FERGUSON-SMITH, M. A. & RATCLIFFE, J. G. (1975) A Semi-automated Serum Alpha-foetoprotein Radioimmunoassay for Prenatal Spina Bifida Screening. *Br. J. Obstet. Gynaec.*, **82**, 718.

VINCENT, R. G., CHU, T. M., FERGEN, T. B. & OBSTRANDER, M. (1975) Carcinoembryonic Antigen in 228 Patients with Carcinoma of the Lung. *Cancer*, **36**, 2069.